# Treatment of Mixture Pollutants with Combined Plasma Photocatalysis in Continuous Tubular Reactors with Atmospheric-Pressure Environment: Understanding Synergetic Effect Sources

**DOI:** 10.3390/ma16216857

**Published:** 2023-10-25

**Authors:** Lotfi Khezami, Aymen Amin Assadi

**Affiliations:** 1 Department of Chemistry, College of Sciences, Imam Mohammad Ibn Saud Islamic University (IMSIU), P.O. Box 5701, Riyadh 11432, Saudi Arabia; 2 College of Engineering, Imam Mohammad Ibn Saud Islamic University (IMSIU), P.O. Box 5701, Riyadh 11432, Saudi Arabia; 3 Univ. Rennes, École Nationale Supérieure de Chimie de Rennes, CNRS, ISCR (Institut. des Sciences Chimiques de Rennes)—UMR 6226, Campus de Beaulieu, Av. du Général Leclerc, 35700 Rennes, France

**Keywords:** mixture of pollutants, coupling system, plasma, photocatalysis, synergetic effect, mineralization

## Abstract

This study investigates the pilot-scale combination of nonthermal plasma and photocatalysis for removing Toluene and dimethyl sulfur (DMDS), examining the influence of plasma energy and initial pollutant concentration on the performance and by-product formation in both pure compounds and mixtures. The results indicate a consistent 15% synergy effect, improving Toluene conversion rates compared to single systems. Ozone reduction and enhanced CO_2_ selectivity were observed when combining plasma and photocatalysis. This process effectively treats pollutant mixtures, even those containing sulfur compounds. Furthermore, tests confirm nonthermal plasma’s in-situ regeneration of the photocatalytic surface, providing a constant synergy effect.

## 1. Introduction

Outdoor air pollutants originate from familiar anthropogenic sources, including industry, transportation, heating, and agriculture [1]. These pollutants have two types of effects: (i) local effects on health and the environment, necessitating short- and medium-term actions, and (ii) global effects on the planet and climate, manifesting in the long term. In response to these concerns, the European directive 2016/2284/EC was published, aiming to reduce national emissions of air pollutants, including NH_3_, SO_2_, NOx, and volatile organic compounds (VOCs) (excluding CH_4_), with aromatic compounds posing the highest health risks [2]. Recognizing the urgency, France pledged 2005 to reduce its NMVOC, NOx, and SO_2_ emissions by 52 to 77% and ammonia by 13% [1,2,3]. Meeting this challenge necessitates the development of advanced treatment processes for industrial effluents, specifically at the emission step, to restrict the concentrations and fluxes released into the environment [4]. Current processes employing a liquid phase and oxido-reduction mechanisms, such as Stretford, Ferrifloc, Sulfurex, Burner-Scrubber, Catalyst-Scrubber, and Ozone processes, rely on large-volume equipment prone to corrosion caused by aggressive solutions [4].

Consequently, these processes incur high investments and maintenance costs, substantial chemical reagent consumption, and pose environmental issues during waste effluent disposal [5]. Thus, treatment processes often fail to meet industry requirements due to cost concerns. Therefore, an alternative process that does not necessitate the use of reagents but only relies on a power supply while generating only mineralizable inorganic by-products (CO_2_, H_2_O, etc.) would align better with industry needs, especially if space requirements and energy consumption are manageable [6].

In recent years, investigations have demonstrated the effectiveness of dielectric barrier discharge (DBD) reactors for hazardous pollutant removal from gas streams with low VOC concentrations at ambient temperatures [7]. The simultaneous reduction in coexisting pollutants has also been studied [8]. Despite its good attributes, DBD plasma has some drawbacks, such as the formation of toxic by-products like CO, NO, NO_2_, and O_3_. Achieving the desired total oxidation of CO_2_ and H_2_O is often challenging [9,10,11,12]. To address these challenges, coupling DBD plasma with photocatalysis presents a ‘zero waste and zero reagent’ technology with the potential for synergy and low energy consumption [13,14]. Several studies have investigated the coupling of DBD plasma and photocatalysis using different reactor types and targeting various odorous compounds, including isovaleraldehyde, isovaleric acid, trimethylamine, ammonia, and dimethyl disulfide (DMDS) [15]. It is known that the presence of a catalyst in the plasma enhances performance [16,17,18,19]. To avoid catalytic surface poisoning and maintain energetic efficiency, producing a higher concentration of reactive species through the controlled adjustment of the pulsed discharge conditions is crucial, as these species have very short lifetimes [18,19,20,21].

This study aims to investigate VOC removal by coupling plasma and photocatalysis, focusing on new electrode configurations, on the performance in handling pollutant mixtures, and on understanding the catalytic surface poisoning mechanisms through tests on the reuse part of the combined system. In fact, the regeneration effect of plasma on the material surface and the understanding of the reactional mechanism in order to lift the scientific barriers was studied in detail in this paper.

## 2. Materials and Methods

### 2.1. Experimental Setup

**Figure 1:**
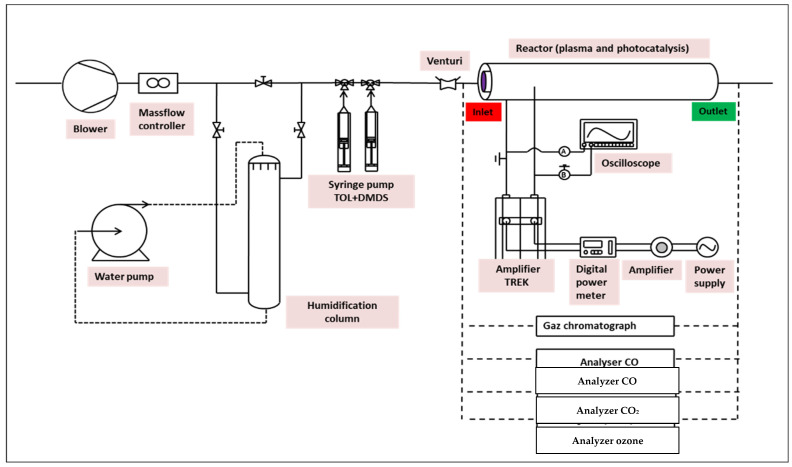
General schematic view of the photocatalysis and non-thermal plasma pilot flowsheeting.

The oxidation (photocatalysis and plasma) runs were conducted using the experimental setup shown in Figure 1. It consists of a tubular cylindrical reactor formed by two concentric Pyrex tubes (100 cm long), one outer tub of 76 mm, and an inner tube of 58 mm. Their wall thickness was about 4 mm. The reactor can be combined with (i) photocatalysis by using an external UV lamp (Philips TL 40W/05 (Philips, Canton of Flayosc, France)) and/or (ii) DBD plasma (Dielectric Barrier Discharge system) by applying high voltage power. Experiments were realized at ambient temperature (25 °C) and atmospheric pressure. A TESTO sensor is used to measure the temperature and relative humidity. Before photocatalytic experiments, the UV lamp (100 cm long, in the inner concentric cylinder) is activated for homogeneous irradiation. The flow rate (a maximum of 10 m^3^.h^−1^) at the system’s inlet is controlled using a mass flow meter (Bronkhorst In-Flow). For humidity experiments (from 5 to 90 ± 5%), a variable part of the airflow is derived through a packed column where water flows in the counter current (Figure 1). Two syringe/syringe driver systems (KD Scientific Model 100) were used continuously for liquid Toluene and DMDS injecting into the gas stream. A heating band was used in the injection zone to achieve a good evaporation of pollutants (Figure 1). 

**Figure 2:**
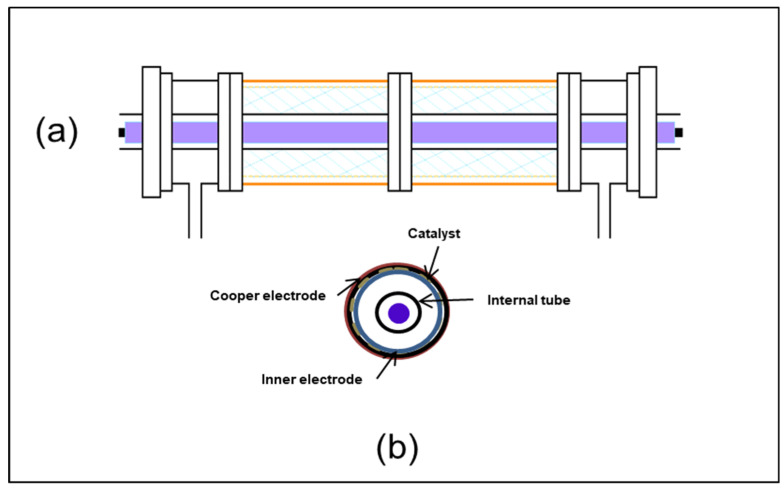

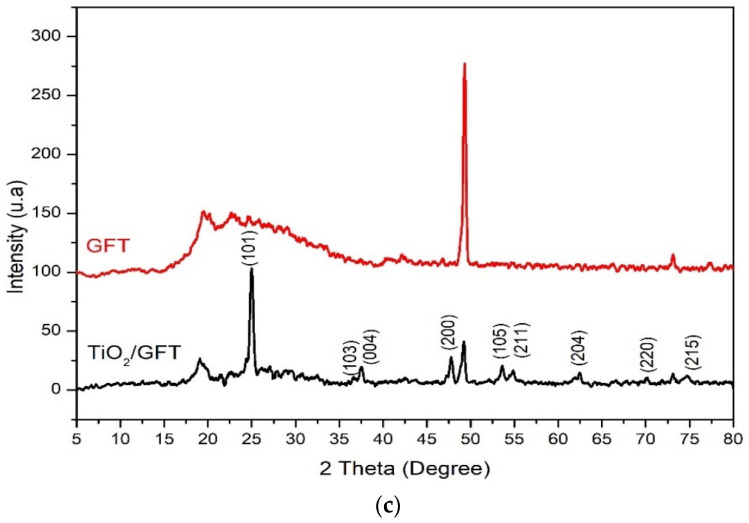
Scheme (**a**) and sectional drawing (**b**) of the cylindrical reactor. (**c**) XRD diffractogram of pristine GFT and GFT coated with TiO_2_.

In the case of DBD plasma equipment, the different experimental parts used to create the plasma are illustrated in Figure 1. Plasma discharge was generated by applying high voltage using a signal generator (BFi OPTILAS (SRS) reference DS 335/1)-USA. The applied tension with sinusoidal waveform was amplified 3000 *v*/*v* using a TREK 30A/40 amplifier-(Denver, CO, USA) [1,2,3,4,5]. The outer and inner electrodes were connected to the amplifier (Figure 2). The voltages applied in the plasma reactor are measured using high-voltage probes and recorded with a digital oscilloscope (Lecroy wave surfer 24Xs, 200 MHz).

The operating parameter and their ranges are summarized in Table 1.

A commercial Glass Fiber Tissue (BET surface of 300 m^2^.g^−1^, 5–10 nm of diameter and 100% Anatase), produced by Ahlstrom Research and Services, was used as a photo-catalyst which contains (i) 13 g.m^−2^ of colloidal silica, (ii) 13 g.m^−2^ of titanium dioxide nanoparticles, and (iii) inorganic fibers. To achieve this catalyst, Ahlstrom Research and Services starts with impregnating glass fibers using SiO_2_ and TiO_2_ nanoparticles suspension in pure water using an industrial-sized press (PC500 Millennium). The second step is a drying step of impregnated fibers [6]. The crystalline phase of the coated photocatalyst on the GFT support was examined using the X-ray diffractometer. The optical band gap of TiO_2_ nanoparticles has a value of 3.2 eV. 

Figure 2c shows the XRD diffraction pattern of the GFT coated with TiO_2_ photocatalyst and the pristine GFT.

The average size of the TiO_2_ nanoparticles was calculated following the Scherrer equation considering the intense (101) plane peak, and the obtained value is 16.33 nm.

### 2.2. Analytical Methods

- The same gas volume (500 µL) was continuously sampled to monitor oxidation phenomena under the photocatalytic plasma reactor. The concentration of Toluene was determined using Gas Chromatography (GC) with a Clarus GC-500 chromatograph equipped with a flame ionization detector (FID) (Salt Lake City, UT, USA) and a 60 m × 0.25 mm polar DB-MS capillary column (film thickness, 0.25 μm). The FID detector was powered by an air and hydrogen mixture (H_2_). Helium (He) was used as the carrier gas at a flow rate of 1 mL.min^−1^. The analysis conditions included injection and detection temperatures of 250 °C for both, and the oven temperature was programmed to maintain 90 °C throughout each analysis (analysis time: 4 min). The analysis method for DMDS sulfur pollutant has been described in detail in our previous work [5].
- CO_2_ measurements were determined using a Fourier Transform Infrared Spectrophotometer (FTIR) from Environment SA (MIR 9000H). The mineralization step was continuously monitored during the oxidation (plasma/photocatalysis) process using a pump system to control the outlet gas stream. For CO analysis, samples were taken using a gas analyzer (NO/CO ZRE marketed by Fuji Electric France S.A.S.). The SO_2_ outlet concentration was measured using a MEDOR gas analyzer (THT MEDOR^®^-Houston, TX, USA).
- The amount of Ozone generated during the oxidation step with plasma was determined using sodium thiosulfate titration. A membrane pump (KNF lab N86k18) delivered part of the flow exits and then bubbled into a potassium iodide solution (KI, at 10^−2^ M). The chemical reaction between KI and Ozone (Equation (1)) resulted in the appearance of a yellow color, which was then neutralized through titration with a sodium thiosulfate solution (Na_2_S_2_O_3_, at 10^−3^ M) until a colorless solution was obtained (Equation (2)) [7]. The titration was carried out in an acid medium by adding concentrated hydrochloric acid (HCl) to the final solution.

O_3_ + 2I^−^ → I_2_ + O_2_ + O^−^ + e^−^ (1)

I_2_ + 2 S_2_O_3_^2−^ → 2I^−^ + S_4_O_6_^2−^ (2)

## 3. Results and Discussion

The experimental parameters are defined as follows:

- (C_inlet_) and (C_outlet_) represent the inlet and outlet concentration of pollutant (mg.m^−3^), respectively.
- The degradation rate of the pollutant with each process (%) = (1 − C_outlet_/C_inlet_) × 100.
- The value of the Synergetic Effect (SE) is calculated using the following expression:

SE = RE_combined process_/[RE_plasma_ + RE_photocatalysis_].

- The Specific Energy SE (J/L) = [P(W)/Q(m^3^.s^−1^)]/1000, where P is the input power in the function of the applied voltage and Q is the flowrate.
- The sulfur dioxide selectivity (SSO_2_) = ([SO_2_]_outlet_ − [SO_2_]_inlet_) × 10^4^/(n_s,cov_ × RE × [C]_inlet_).
- The carbon dioxide and monoxide selectivities (SCO_x_) = ([CO_x_]_outlet_ − [CO_x_]_inlet_) × 10^4^/(n_c,cov_ × RE × [C]_inlet_), where [C] is the concentration of DMDS/Toluene and n_c,VOC_ represents the number of carbons in the molecules (two for DMDS and seven for Toluene).

The degradation studies of (i) Toluene alone (100% C_7_H_8_), (ii) DMDS alone (100% C_2_H_6_S_2_) on a continuous annular reactor, and (iii) their binary mixture (Toluene 50%-DMDS 50%) were investigated. The experiments with the photocatalysis process were carried out under different operating conditions. The plasma process performance was also monitored separately from the photocatalysis (without external UV-lamp), and then the association of the photocatalysis/plasma was studied.

### 3.1. Photocatalysis Treatment: (i) Effects of Initial Pollutant Concentration and Air Flow on Degradation and (ii) Effect of Water Vapor

This study systematically investigated the impact of varying initial toluene concentrations (10 and 20 mg.m^−3^) and airflow rates (1–4 m^3^.h^−1^) on the efficiency of the photocatalytic removal of toluene. The experiments were conducted with and without a light source (UV-lamp OFF) during an initial adsorption step to ensure stable inlet toluene concentrations. It was observed that, as the gas flow supplying the photocatalytic reactor was increased, the efficiency of toluene degradation was decreased. This trend can be attributed to the shorter contact time between toluene molecules, active sites on the catalyst, and oxidation species at higher flow rates, resulting in reduced degradation efficiency. The experimental data indicated that toluene degradation was approximately 38.5% at 1 m^3^.h^−1^ for an initial concentration of 10 mg.m^−3^ but decreased to around 13% at 4 m^3^.h^−1^. Moreover, an increase in the initial toluene concentration also led to reduced oxidation performance, with degradation rates of 38.5% and 21.9% observed at 1 m^3^.h^−1^ for initial concentrations of 10 and 20 mg.m^−3^, respectively. This behavior aligned with previous research on TiO_2_-based catalysts [1,6,7,8,9]. 

**Figure 3:**
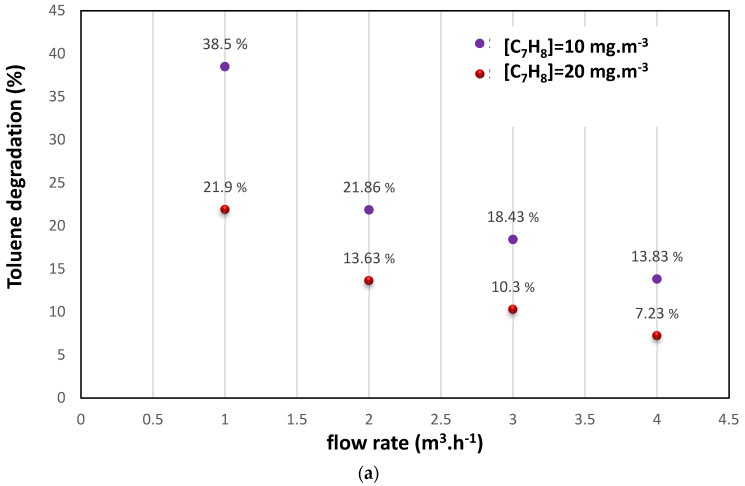

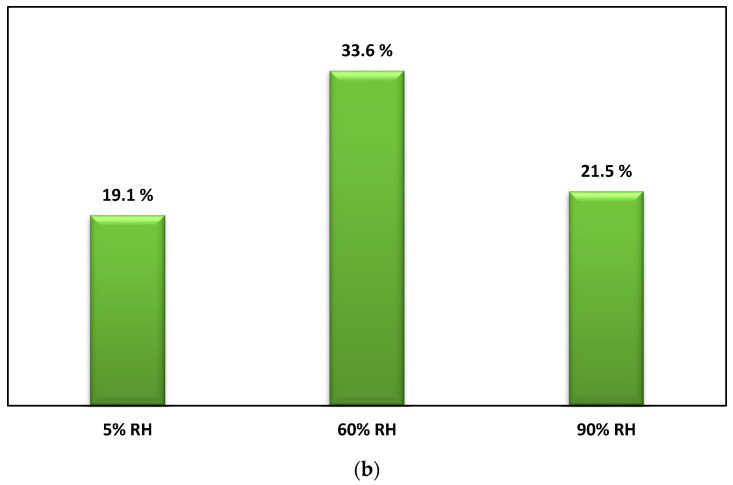
(**a**) Toluene performance degradation at different [C_7_H_8_] and flow rates in the photocatalysis process. [T = 20 °C, UV_intensity_ = 20 W m^−2^]. (**b**) Toluene performance degradation at different rates of humidity in the photocatalysis process. [Flow rate = 2 m^3^ h^−1^, [C_7_H_8_] = 10 mg m^−3^, T = 20 °C, UV_intensity_ = 20 W m^−2^].

The influence of humidity levels was also investigated by maintaining a constant inlet toluene concentration (10 mg.m^−3^) and an airflow rate of 2 m^3^.h^−1^ while varying humidity levels at approximately 5%, 60% ± 5, and 90% ± 5 using a humidification column. Two distinct behaviors in toluene removal were revealed in Figure 3b. At lower humidity levels (<60% ± 5), an improvement in toluene removal was observed due to active intermediate species generated under these conditions, enhancing the oxidation step and overall photocatalytic toluene removal. Favorable toluene removal efficiency was demonstrated in the experiments at approximately 60% ± 5 humidity levels, increasing efficiency from 19 to 33.6%. However, at higher humidity levels (>60–90%), competitive adsorption between water vapor and toluene molecules on active sites became more pronounced, decreasing toluene removal efficiency. At high humidity levels (90% ± 5), a slight decrease in toluene removal efficiency to 21.5% was observed. The significance of humidity as an experimental parameter in photocatalytic oxidation processes is highlighted by our comprehensive analysis, with lower humidity favoring toluene removal and higher humidity exerting a negative impact. Thus, increasing the relative humidity inside the reactor results in a net presence of water molecules. The water molecules adsorbed on the surface of the photocatalyst result in photogenerated holes following oxidation leading to the formation of OH radicals known as reactive species in the photocatalytic air treatment. On the other hand, the significant presence of water vapor molecules at high relative humidity levels reverses the trend and reduces the conversion of the pollutant due to the phenomenon of competition between the water molecule and the adsorption of ethylbenzene on the active sites of the photocatalyst [5].

### 3.2. DBD Direct Plasma Treatment: Effect of Plasma Energy

**Figure 4:**
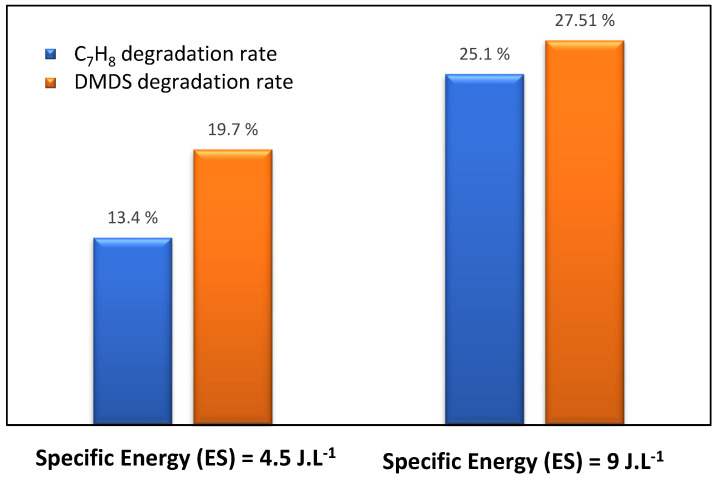
Toluene/DMDS performance degradation at different plasma specific energy SEs in the plasma process. [Q = 2 m^3^ h^−1^, [COV] = 14 ppm, RH = 55%, T = 20 °C].

To study the performance of pollutants’ (toluene and DMDS) degradation via a plasma reactor, the degradation study of toluene was performed with (i) humid airflow (2 m^3^.h^−1^, 55% of humidity), (ii) an inlet concentration of 14 ppm, and (iii) a plasma energy of 4.5 J.L^−1^ and 9 J.L^−1^. The same methodology was applied to DMDS, where the reactor contained a similar pollutant concentration. Figure 4 shows the degradation rate of toluene/DMDS studied separately at different plasma energies. The experimental data via DBD plasma configuration indicate that, with the two pollutants (aromatic and sulfuric compounds), the increase in specific energy (plasma power) leads to an increase in the removal efficiency of contaminants [7,8,9]. In our previous work, a similar trend of removal efficiency was displayed in fatty acids [10], aldehydes [15], and amines [21], either on a pilot or on an industrial scale. We reported that the removal efficiencies of these molecules strongly depend on the applied voltage. It was observed that increasing energy enhances the level of electrons, which improves the reactive oxygen species formation and consequently leads to greater removal efficiency. In our study, the toluene removal improved from 13% to 25.1% with 4.5 J.L^−1^ and 9 J.L^−1^ of plasma power, respectively. As for DMDS, when the specific energy amount is more and more important (9 J.L^−1^), the DMDS rate (27.51%) is slightly higher than for toluene (25.1%).

### 3.3. Treatment by Coupling Process (Photocatalysis/Plasma): Comparison of Process Performance

**Figure 5:**
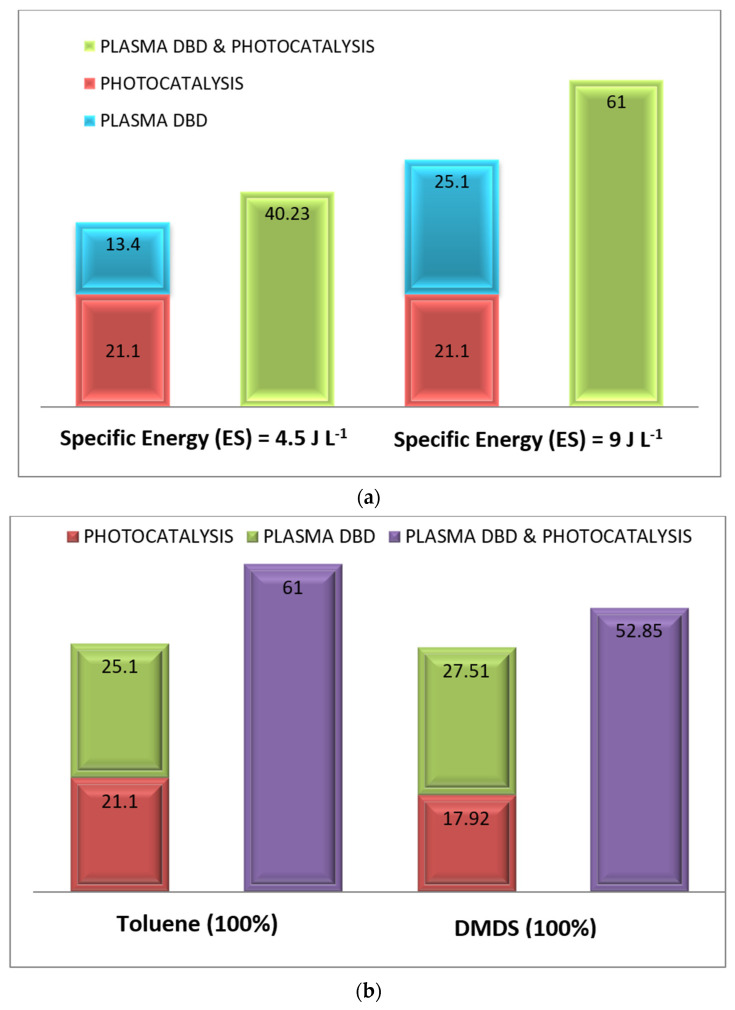
(**a**) Toluene performance degradation at different plasma specific energy SE via photocatalysis, plasma, and their combination: Evidence for synergetic effect. [Q = 2 m^3^ h^−1^, [toluene] = 14 ppm, RH = 50%, T = 20 °C, UV_intensity_ = 20 W m^−2^]. (**b**) Pollutant performance degradation when applying photocatalysis, plasma, and their combination: Evidence for synergetic effect. [Q = 2 m^3^ h^−1^, [pollutant alone: 100% toluene or DMDS] = 14 ppm, ES= 9 J L^−1^, RH = 50%, T = 20 °C, SE= 9 J/L, UV_intensity_ = 20 W m^−2^].

The oxidation of toluene was monitored via three processes: (i) photocatalysis, (ii) DBD plasma, and (iii) a combination of photocatalysis and plasma at different plasma energies (4.5 and 9 J.L^−1^). In this section of the study, these two methods ((i) and (ii)) were used separately and simultaneously (iii) to enhance the degradation performance of the process. Comparing the treatment with the simultaneous application (photocatalysis/plasma) to that with photocatalysis and plasma applied separately, the experimental data (Figure 5a) indicate that the coupling exhibits a higher performance than the sum of photocatalysis alone and plasma alone. At a plasma energy of 9 J.L^−1^, the combined process achieved a toluene degradation rate of 61%, while the sum of the degradation rate for photocatalysis and plasma separately was 46.2%. Similarly, at low energy (4.5 J.L^−1^), the toluene degradation reached 40.23% when the combined application was used, compared to 34.5% for the sum of both processes used alone. In this case, an enhancement of 10% ± 3 in the removal efficiency was observed. The same methodology was applied to DMDS (see Figure 5b). The results indicate a strong synergy between both processes for any pollutant used. Plasma significantly contributes to the desorption of the degraded by-products adsorbed on the TiO_2_ surface through the active species (O_2_°^−^, O°, HO°), leading to the increased catalytic activity of the photocatalyst (in our case, TiO_2_ coated on Glass Fiber Tissue) and improved photocatalytic degradation [6,21,22,23,24,25,26,27]. For DMDS, the degradation rate during the combined application of photocatalysis and plasma (53%) surpasses the rates achieved with plasma or photocatalysis alone (45.4%). The same behavior has been found by Qi and his coworkers, with toluene removal in a plasma–catalytic hybrid system over Mn-TiO_2_ and Fe-TiO_2_ [28]. Moreover, Wang and his collaborators highlighted the synergetic effect on CO_2_ reduction in the presence of Dual-plasma enhanced 2D/2D/2D g-C_3_N_4_/Pd/MoO_3_ [11].

The combination of these processes demonstrates the synergy between DBD plasma and photocatalytic oxidation [13,16,17,18,19,20], which can be attributed to the following:

(i). The action of plasma radicals (N°, O°, H°, OH°), renewing the catalytic site and improving the removal/mineralization step;
(ii). The contribution of the reactive species generated by plasma to photocatalytic mechanisms;
(iii). The Ozone and UV-light reactions generate highly reactive radicals that can activate TiO_2_ and enhance performance;
(iv). The enhancement of mass transfer of pollutants by the ionic wind generated by plasma;
(v). The in-situ regeneration of the catalytic surface in the presence of the micro discharge of plasma.

### 3.4. Treatment by Coupling Process (Photocatalysis/Plasma): Effect of Mixture (Toluene/DMDS) and Plasma Power

**Figure 6:**
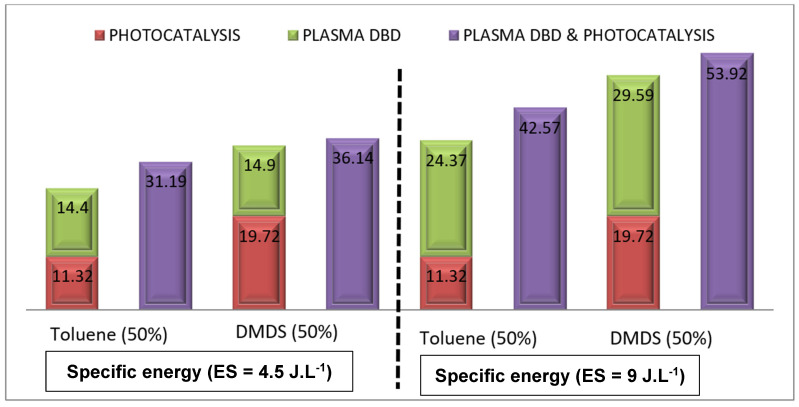
Evolution of toluene/DMDS degradation rate in mixtures at different plasma specific energy SE via photocatalysis, plasma, and their combination: Evidence for synergetic effect. [Q = 2 m^3^ h^−1^, [Mixture of pollutant: 50% toluene + 50% DMDS] = 14 ppm, RH = 50%, T = 20 °C, UV intensity = 20 W m^−2^].

The same methodology was applied to the mixture of toluene and DMDS. Figure 6 illustrates the removal efficiency of the toluene/DMDS mixture using three processes: (i) photocatalysis, (ii) DBD plasma, and (iii) photocatalysis/plasma at different plasma energies. For the mixture studied, the reactor was supplied with: a humid air flow (2 m^3^.h^−1^, 55% humidity), an inlet concentration of 14 ppm ([Toluene] = [DMDS] = 7 ppm), and plasma energies of 4.5 and 9 J.L^−1^. As depicted in Figure 6, plasma energy is a crucial parameter for monitoring plasma application and it can significantly impact degradation efficiency.

In the case of the mixture with high energy (9 J.L^−1^), the results indicate that (i) toluene degradation reached 42.57% when both applications were combined, compared to 35.69% when the two applications were applied separately, and (ii) DMDS degradation reached 53.92% when both applications were combined, compared to 49.31% for separate applications. Comparing the treatment of the toluene/DMDS mixture to the separate treatment of toluene and DMDS, the experimental data (Figure 6 and Figure 5b, respectively) demonstrate that the coupling exhibits higher removal efficiency, surpassing the sum of photocatalysis and plasma treatments when applied separately.

The toluene/DMDS mixture results indicate a more significant synergetic effect for DMDS degradation than for toluene. When photocatalysis/plasma were used together, 42.57% of the toluene in the mixture and 53.92% of the DMDS in the mixture were decomposed. In contrast, during single treatments (Figure 5b), toluene removal reached 61%, and 52% of the DMDS was decomposed. This different behavior can be attributed to the competitive adsorption/oxidation of the two pollutants in the mixture, which becomes more pronounced [22,23,24,25]. The molecular chain structure of DMDS (C_2_H_6_S_2_) hampers the adsorption/oxidation and, thus, the photocatalytic oxidation of toluene (C_7_H_8_). This observation is consistent with the findings reported by Assadi et al. [21], who demonstrated a decrease in oxidation performance (in the case of a VOC mixture) due to the competitive interactions between pollutants, by-products, and active sites.

### 3.5. Study of By-Products Generation: Ozone Selectivity of CO/CO_2_/SO_2_

#### 3.5.1. Monitoring of the Ozone Formed

**Figure 7:**
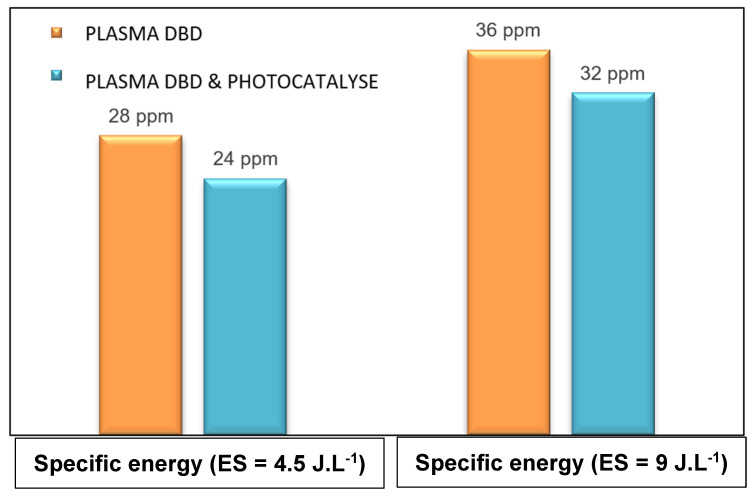
Evolution of ozone concentration (ppm) with plasma specific energy SE. [Q = 2 m^3^ h^−1^, [Mixture of pollutant: 50% toluene + 50% DMDS] = 14 ppm, RH = 50%, T = 20 °C, UV intensity = 20 W m^−2^].

Ozone formation, a strong oxidizing by-product, occurs during the operation of the DBD-plasma/photocatalytic reactor. Extensive experiments on oxidation under (i) a DBD-plasma reactor or (ii) coupling plasma/photocatalysis technology have been conducted and detailed in our previous research studies [1,4,6,7,9,23,24,25,26,27]. These studies have shown that increasing the energy of plasma leads to generating a significant amount of ozone in the exhaust. However, experiments with the DBD plasma/photocatalysis combination have been performed to mitigate the excessive ozone production at low plasma energies (4.5 and 9 J.L^−1^). The humid air flow was fixed at 2 m^3^.h^−1^ with 55% humidity, and the concentration of toluene/DMDS was maintained at 14 ppm. UV irradiation tests (with lamp ON/OFF) were conducted to monitor ozone formation at the reactor outlet at various plasma energies. The results, depicted in Figure 7, illustrate the behavior of ozone during the operation of DBD plasma technology and the coupling of plasma/photocatalysis.

A slight decrease in ozone concentration was observed (Figure 7) with values of 36 and 32 ppm for (i) plasma alone and (ii) plasma/photocatalysis, respectively. This decrease can be attributed to the decomposition of ozone through reactions (3), (4), and (5), facilitated by the photo-generated radicals (H°, HO°) [26]. It is important to note that adding external UV light to the DBD-plasma system (coupling plasma with UV lamp ON) can play a crucial role in promoting ozone degradation into highly reactive species (O2°^−^ and HO_2_°), thereby significantly enhancing the oxidation step.

(3) $$H_{2}O+e^{-}\to H{}^{\circ}+OH{}^{\circ}+e^{-}$$

(4) $$O_{3}+OH{}^{\circ}{\to O}_{2}+{HO}_{2}{}^{\circ}$$

(5) $$O_{3}+H{}^{\circ}{\to O}_{2}+OH{}^{\circ}$$

#### 3.5.2. CO_2_, SO_2_, CO Selectivity

**Figure 8:**
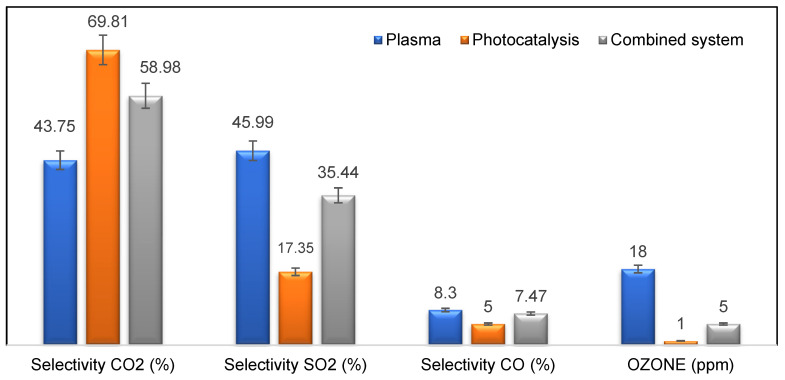
Variation in CO_2_, SO_2_, and CO (%) and ozone (ppm) during toluene/DMDS removal with photocatalysis/plasma coupling. [Q = 2 m^3^ h^−1^, [Mixture] = 14 ppm, RH = 50%, T = 20 °C, UV_intensity_ = 20 W m^−2^, SE = 4.5 J L^−1^].

In this investigation, the selectivity rates of carbon dioxide (CO_2_), sulfur dioxide (SO_2_), and carbon monoxide (CO) under different oxidation conditions were analyzed. Figure 8 presents the experimental data, showcasing distinct selectivity rates for these pollutants. Carbon dioxide (CO_2_) exhibited the highest selectivity rate among the three, achieving 69.81% through photocatalysis alone, 43.75% through plasma alone, and a further increase to 58.98% when plasma and photocatalysis were combined. On the other hand, sulfur dioxide (SO_2_) showed a selectivity rate of 17.35% through photocatalysis, while plasma alone achieved 46%. However, the combination of plasma and photocatalysis resulted in a decreased selectivity rate compared to CO_2_. For carbon monoxide (CO), selectivity rates were negligible, with only 7% of the CO mineralization rate observed through photocatalysis, plasma, and plasma/photocatalysis.

It is worth noting that ozone (O_3_) concentrations decreased compared to plasma alone. Previous research has shown that incorporating external UV light into the DBD–plasma system (via coupling plasma with UV lamp ON) can significantly enhance the mineralization rate and improve the degradation of pollutants and by-products [16,17,18,19,20].

## 4. Reusability and In Situ Regeneration

A series of experiments consisting of four cycles was conducted to evaluate the photocatalytic stability of the catalyst after multiple cycles. These experiments involved alternating phases: (i) the oxidation step using photocatalysis, plasma, and the coupling of both, and (ii) the catalyst regeneration step. The photoactivity tests were conducted under dry conditions with a continuous flow rate of 2 m^3^.h^−1^, using a toluene/DMDS mixture with a concentration of 14 ppm.

**Figure 9:**
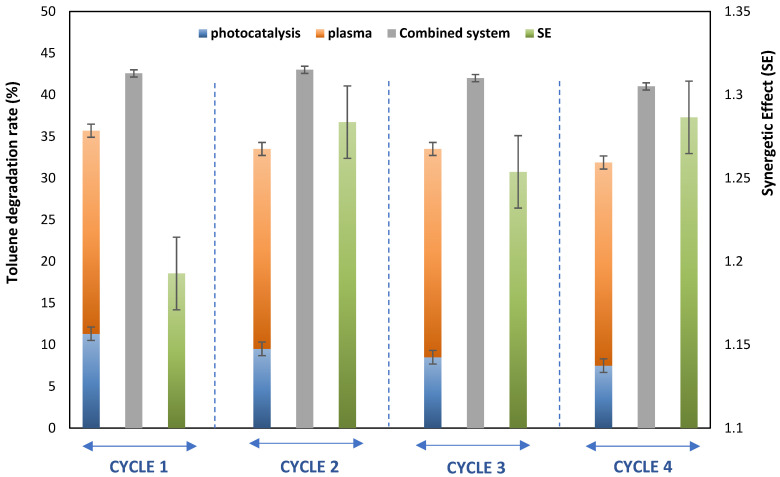
Effect of catalysis reusability on synergetic effect in the case of mixture toluene/DMDS. [Q = 2 m^3^ h^−1^, [Mixture] = 14 ppm, RH = 50%, T = 20 °C, UV_intensity_ = 20 W m^−2^, SE = 4.5 J L^−1^].

Figure 9 presents the degradation rate of toluene in the mixture and the synergetic effect (SE) value after four cycles (each cycle consisting of experiments with photocatalysis, plasma, and their combination). It can be observed that the degradation rate of toluene slightly decreases after four cycles of continuous oxidation, resulting in a 10% loss of the catalyst’s photoactivity. However, a stable degradation rate is observed when the coupling of plasma/photocatalysis is applied. In our case, the regeneration step involved using photocatalysis/plasma.

Previous studies have also observed the deactivation of photocatalysts during the oxidation of sulfur compounds [27]. These studies have shown that plasma can effectively regenerate poisoned catalysts. Therefore, the results indicate that the regeneration process can be enhanced by combining photocatalysis and plasma [28,29,30,31,32,33,34]. This finding confirms the in situ regeneration of the photocatalytic support in the presence of plasma [35,36,37,38,39,40,41].

## 5. Conclusions

In this comprehensive study, we conducted a thorough investigation into various parameters, including the synergetic effect (SE), inlet concentrations of toluene (TOL) and DMDS, and relative humidity (RH), to assess their influence on the performance of three distinct processes: DBD plasma, photocatalysis, and the combined DBD plasma/photocatalysis system. Our findings have illuminated the pivotal role of water vapor in VOC removal, revealing optimal RH values that enhance CO_2_ selectivity and diminish CO formation. Furthermore, RH was observed to have a mitigating effect on ozone formation.

Across all operational parameters explored, it is evident that coupling DBD plasma with a TiO_2_ catalyst under external UV irradiation can yield a synergetic effect, resulting in improved toluene and DMD_S_ removal. The significant enhancement in CO_2_ selectivity during the coupling process is particularly noteworthy, a remarkable 11% improvement compared to DBD plasma alone. The observed reduction in ozone concentration during plasma–photocatalysis coupling can be attributed to the breakdown of ozone into more active oxidizing species facilitated by UV radiation.

These findings hold profound practical implications for pollutant removal and treatment strategies. As demonstrated in this study, the combination of DBD plasma and photocatalysis presents a promising avenue for more efficient and environmentally friendly approaches to addressing VOC pollution. By unraveling the intricate interplay of parameters and processes, we are better positioned to develop cleaner and more sustainable solutions for industrial effluent treatment.

Future research endeavors could delve deeper into optimizing the coupling process, explore additional parameters, and investigate its adaptability in diverse industrial settings. The pursuit of innovative technologies that reduce environmental impact while aligning with industry requirements remains of paramount importance.

## Figures and Tables

**Table 1:** Parameters of combined reactor.

Parameter	Value/Domain
Gas temperature	Ambient (293 K)
Gas pressure	Atmospheric pressure (1 atm)
Relative humidity	(5, 60, 90) ± 5%
Specific Energy (SE)	4.5–9 J.L^−1^
Target compound concentartion	10–60 mg.m^−3^
Residence time	1.36 s

## Data Availability

Not applicable.
